# Surgical management of tumor-mimicking posteriorly migrated lumbar disc fragment using a quadrant minimally invasive approach: a case report and literature review

**DOI:** 10.3389/fsurg.2026.1822299

**Published:** 2026-06-23

**Authors:** Saifei Meng, Longxiao Wu, Zizu Li, Haolin Tang, Chunlei Liu

**Affiliations:** Department of Spinal Surgery, Qingyuan Hospital Affiliated to Guangzhou Medical University (Qingyuan People's Hospital), Qingyuan, Guangdong, China

**Keywords:** extradural spinal lesions, lumbar disc herniation, minimally invasive surgery, posterior disc migration, tumor-mimicking lesion

## Abstract

**Background:**

Posterior epidural migration of disc fragments (PEMDF) is a rare condition that can mimic spinal tumors on imaging, particularly neurogenic lesions, thereby posing a diagnostic challenge preoperatively. Owing to its atypical posterior extradural location and characteristic contrast enhancement patterns, it is frequently misinterpreted as a neoplastic process. Previous studies have reported that such lesions are commonly managed with open posterior decompression or conventional laminectomy to establish a definitive diagnosis and relieve neural compression. However, reports describing minimally invasive management, particularly the Quadrant approach, remains sacrce. Herein, we present this case to emphasize the diagnostic pitfalls and to demonstrate the feasibility of minimally invasive surgical treatment.

**Case presentation:**

A 67-year-old man presented with a 6-month history of intermittent low back pain, accompanied by progressively worsening bilateral lower-extremity numbness and weakness. Magnetic resonance imaging demonstrated a posterior extradural mass at the L2/3 level, characterized by heterogeneous hyperintensity on T2-weighted images and peripheral rim enhancement following contrast administration. A neurogenic tumor was initially suspected based on these findings. Given the progressive neurological deterioration and diagnostic uncertainty, the patient underwent surgical intervention via a minimally invasive Quadrant approach, achieving complete excision of the lesion. Histopathological examination revealed findings consistent with degenerative intervertebral disc tissue. Postoperatively, the patient showed marked symptomatic improvement, with complete recovery of lower-extremity muscle strength. No neurological deficits or radiological recurrence were observed during the 6-month follow-up period.

**Conclusions:**

PEMDF can closely mimic spinal tumors on magnetic resonance imaging, particularly when peripheral rim enhancement and posterior extradural localization are observed. Awareness of this entity is essential to prevent misdiagnosis. While surgical treatment has traditionally relied on open decompression for both diagnostic confirmation and symptom relief, minimally invasive approaches such as the Quadrant technique, may provide an effective alternative in selected cases. Surgical exploration remains a reliable approach for establishing a definitive diagnosis and providing treatment when imaging findings are inconclusive.

## Introduction

PEMDF is an uncommon presentation of lumbar disc herniation and represents a well-recognized diagnostic pitfall (1). Because the fragment lies posterior to the thecal sac and may exhibit peripheral rim enhancement following contrast administration, it can closely mimic spinal tumors—especially neurogenic tumors—or other epidural space-occupying lesions on magnetic resonance imaging (2). Consequently, the diagnosis is frequently established only during surgery, where excision not only relieves neural compression but also provides tissue for histopathological confirmation (3).

The existing literature consists largely of case reports, small series, and narrative or systematic reviews that emphasize two recurring themes: first, the substantial imaging overlap between posteriorly migrated disc fragments and neoplastic or infectious epidural pathologies; and second, the central role of surgical exploration in clinically significant or diagnostically ambiguous cases (4). Larger syntheses of published cases have shown that posterior decompression—most commonly laminectomy or hemilaminectomy—remains the predominant operative strategy to safely access and remove the migrated fragment and to confirm the diagnosis (4). Individual reports further indicate that even when preoperative imaging strongly suggests a tumor, early surgical intervention is often warranted in the presence of progressive neurological symptoms, with favorable neurological recovery following complete fragment removal (5).

Although surgery is the mainstay for symptomatic PEMDF, conservative treatment—including analgesics, anti-inflammatory drugs, physical therapy, and close monitoring—can be used in selected patients without severe neurological deficits, with rare spontaneous regression (6). However, it is not recommended for those with cauda equina syndrome, progressive weakness, or diagnostic uncertainty, to avoid irreversible nerve injury. Surgery enables rapid decompression and definite pathological diagnosis, while minimally invasive techniques reduce trauma and complications.

With the increasing adoption of minimally invasive spine surgery, posterior minimally invasive approaches have been increasingly applied in the treatment of lumbar disc disease, aiming to minimize soft-tissue disruption while achieving adequate neural decompression (7). Nevertheless, due to diagnostic uncertainty in cases of PEMDF, surgical intervention appears to be the optimal approach for establishing a definitive diagnosis. Although open posterior decompression remains the standard approach, reports of minimally invasive techniques - particularly using the Quadrant tubular retractor - are limited. We therefore present the first case of successful Quadrant MIS excision of a tumor-mimicking PEMDF, demonstrating feasibility, safety, and rapid neurological recovery.

## Case presentation

A 67-year-old male farmer presented with a 6-month history of intermittent low back pain that exacerbated by, prolonged sitting or standing. He also developed neurogenic intermittent claudication occurred after walking approximately 300 meters. During the 2 months prior to admission, he experienced progressive numbness in both thighs and buttocks, which was more pronounced on the left side. He reported occasional mild bilateral lower-limb discomfort 30 years previously but had not received systematic clinical evaluation. Acupuncture and a 5-day course of celecoxib (120 mg/day) prescribed at a local clinic yielded no symptomatic relief. Neurological examination revealed sensory deficits over the posterolateral aspects of both thighs, a positive left sided straight leg raise test, and diminished bilateral knee-jerk reflexes. Manual muscle testing showed left lower limb muscle strength of 4+/5 and 5/5 on the right side. His visual analog scale (VAS) pain score was 7/10.

**Figure 1:**
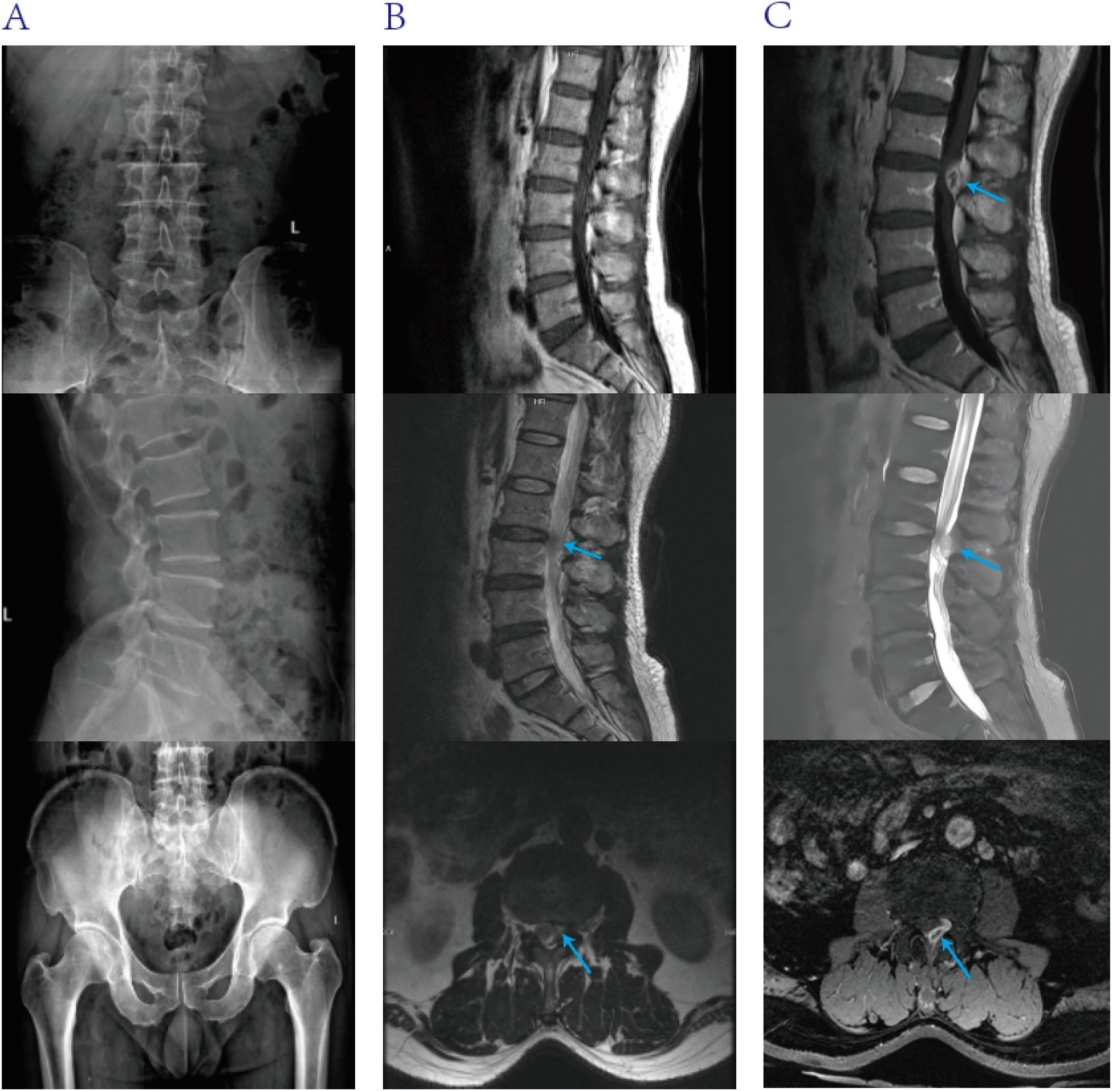
Preoperative radiographic and MRI evaluation. **(A)** Standing anteroposterior and lateral radiographs of the lumbar spine, along with an anteroposterior pelvic radiograph, showing degenerative changes without obvious structural abnormalities. **(B)** Non-contrast MRI sequences. From top to bottom: sagittal T1-weighted image, sagittal T2-weighted image, and axial T1-weighted image. A posteriorly migrated extradural lesion at the L2/3 level is indicated by blue arrows. **(C)** Contrast-enhanced MRI sequences. From top to bottom: sagittal T1-weighted image with gadolinium enhancement, sagittal T2-weighted image, and axial post-contrast T1-weighted image. The lesion demonstrates irregular peripheral enhancement and causes rightward displacement of the dural sac (blue arrows).

To establish a definitive diagnosis, the patient underwent further comprehensive evaluation following admission. Lumbar and pelvic radiographs (Figure 1A) showed no overt structural abnormalities other than degenerative changes. Routine laboratory examinations, including complete blood count, serum C-reactive protein, serum electrolytes, and erythrocyte sedimentation rate, were all within normal reference ranges, thus excluding an infectious etiology. Lumbar spine magnetic resonance imaging (MRI) demonstrated a heterogeneous lesion at the L2/3 level, which as hypointense on T1-weighted images and hyperintense on T2-weighted images (Figure 1B), with rightward displacement of the dural sac. Contrast-enhanced MRI (Figure 1C) revealed irregular peripheral enhancement, raising a strong suspicion of schwannoma.

**Figure 2:**
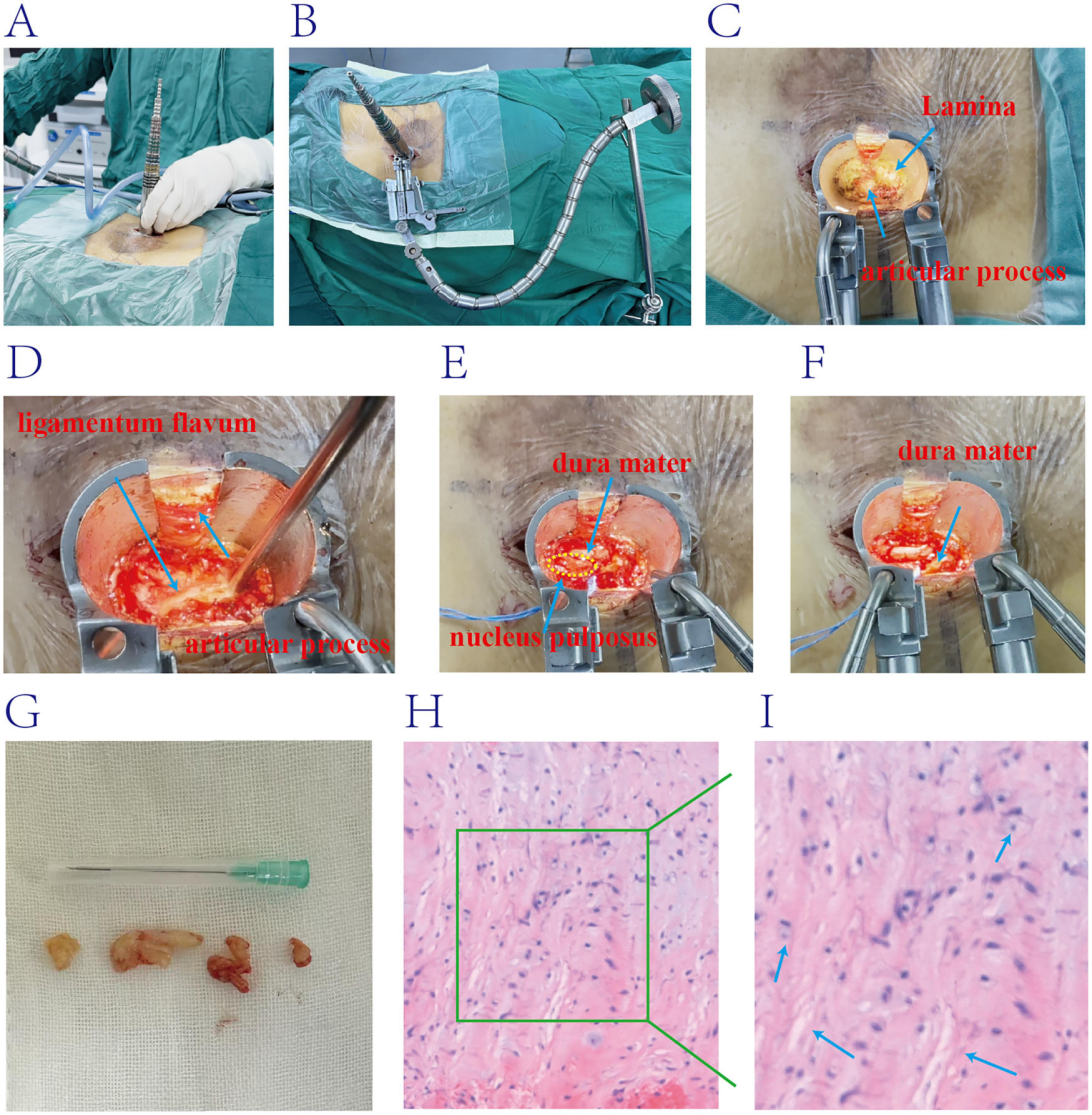
Intraoperative Findings and postoperative histopathological examination. **(A,B)** Establishment of the working channel using a minimally invasive approach.**(C)** Soft tissue dissection to expose the underlying bony structures, including the lamina and articular process. **(D)** Partial laminotomy performed to expose the ligamentum flavum. **(E)** After removal of the ligamentum flavum, the dura mater is visualized, and the herniated nucleus pulposus fragment is identified. **(F)** Complete removal of the herniated disc material, with clear exposure of the dura mater. **(G)** Gross specimen after complete excision, consisting of multiple fragments of soft, yellowish-brown fibrocartilaginous tissue.**(H,I)** Histopathological examination with Hematoxylin and Eosin staining (H&E) demonstrating degenerative fibrocartilaginous tissue, characterized by disorganized collagen fibers, decreased cellularity, and nuclear pyknosis. These findings are consistent with displaced intervertebral disc material.

**Figure 3:**
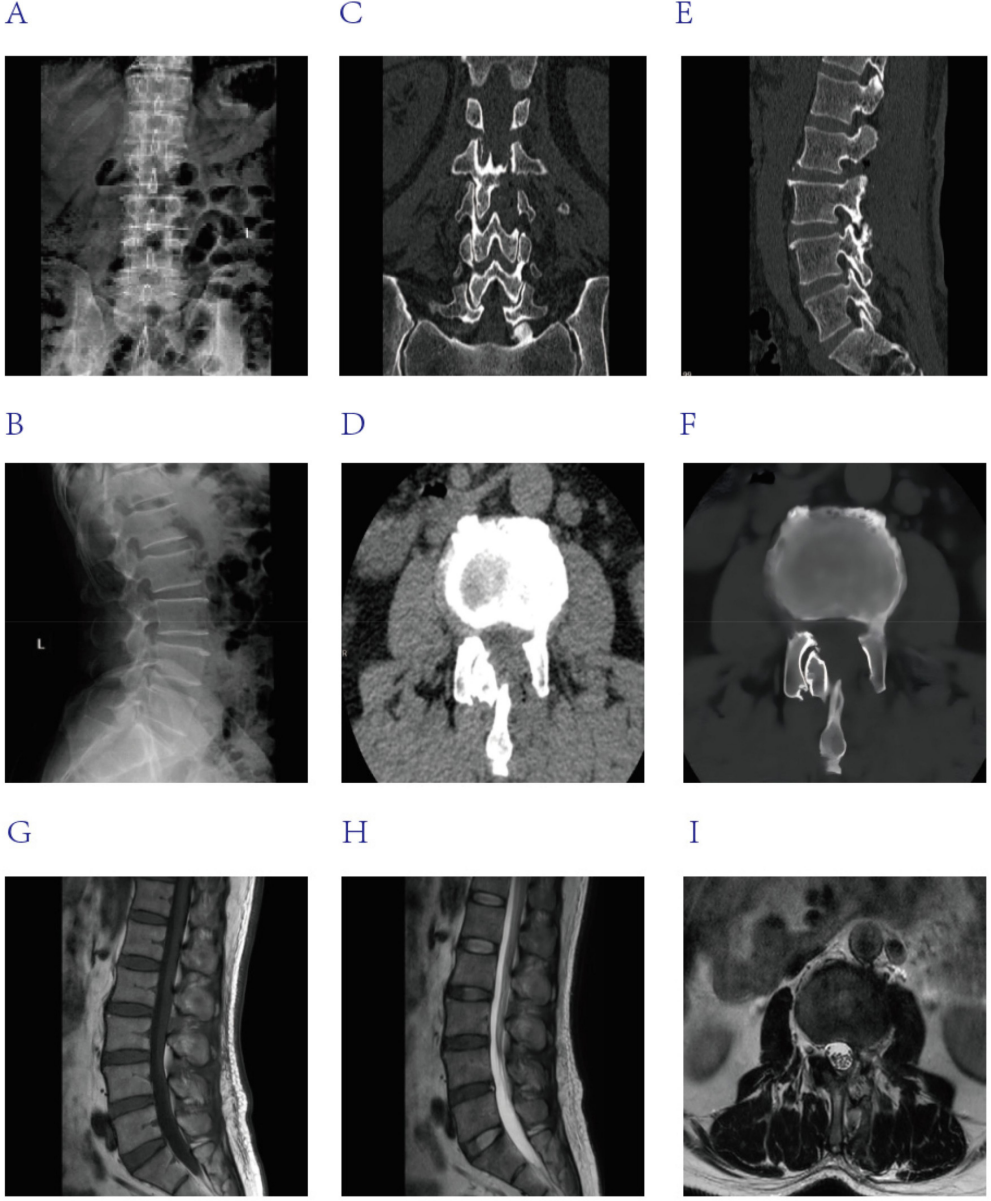
Postoperative follow-up imaging evaluation. **(A,B)** Postoperative lumbar spine hyperextension anteroposterior and lateral radiographs, demonstrating good spinal alignment and stability, with no evidence of abnormal motion. **(C–F)** Postoperative lumbar CT scans, including coronal, sagittal, and axial reconstructions, showing only limited partial laminectomy with well-preserved osseous structures, indicating minimal surgical trauma. **(G–I)** Postoperative lumbar MRI scans, including sagittal and axial sequences, confirming complete resection of the lesion, adequate spinal canal decompression, and no evidence of residual or recurrent disease.

Given the patient's progressive neurological deficits and inconclusive imaging results, minimally invasive resection was performed via the Quadrant tubular retractor system (Figures 2A–F). Intraoperatively, a soft, encapsulated mass compressing the L3 nerve root was identified and totally resected. The cut surface of the lesion showed a fatty-fibrous appearance (Figure 2G). Histopathological examination confirmed degenerative fibrocartilaginous tissue, consistent with sequestrated intervertebral disc material (Figures 2H,I). Postoperatively, the patient achieve significant symptomatic relief, with complete recovery of normal lower limb muscle strengh. At the 6-month follow-up, no recurrence of symptoms or neurological deficits was noted (Figure 3).

## Discussion

Lumbar disc herniation typically occurs via annular rupture, allowing nucleus pulposus material to migrate into the epidural space under the axial loading and repetitive mechanical stress. In most cases, disc fragments migrate anteriorly or laterally, however, posterior epidural migration represents a rare and atypical pattern. Several anatomical and biomechanical factors have been proposed to explain this phenomenon. The posterior longitudinal ligament, peridural membrane, epidural fat, nerve roots, and venous plexus normally serve as natural barriers against posterior displacement of disc material. Disruption or congenital weakness of these structures, combined with high intradiscal pressure, may permit disc fragments to traverse these barriers and reach the dorsal epidural space (6, 8). Once posterior migration occurs, the displaced disc fragment may undergo inflammatory and degenerative changes, resulting in granulation tissue formation and peripheral neovascularization. These changes account for the characteristic rim enhancement observed on contrast-enhanced MRI, which is the first-choice diagnostic modality and closely resembles the imaging features of epidural neoplasms, particularly nerve sheath tumors (8–10). This tumor-mimicking appearance explains why posteriorly migrated disc fragments are frequently misdiagnosed preoperatively and why definitive diagnosis is often achieved only through surgical exploration and histopathological examination (3, 6). According to a comprehensive literature review of 111 cases (6), posterior epidural migration of disc fragment (PEMDF) is an extremely rare condition. It predominantly affects middle-aged men, with the most common level being L3–L4. The main clinical manifestations include cauda equina syndrome, radiculopathy, and low back pain. Most cases require surgical treatment, while conservative management is only suitable for highly selected patients.

As posterior epidural migration of disc fragments is rare and often radiologically indistinguishable from neoplastic lesions,no standardized treatment algorithm has been established. Conservative management has occasionally been reported, particularly in patients without progressive neurological deficits, in whom spontaneous symptom resolution or radiological regression may occur (3). However, such cases are exceptional and primarily involve cervical or other atypical presentations rather than lumbar posterior epidural migration. In contrast, the majority of reported lumbar cases have been treated surgically. Surgical intervention serves a dual purpose: to decompression neural elements and to establish a definitive diagnosis when imaging findings are inconclusive (11). Open posterior decompression, including laminectomy or hemilaminectomy, has historically been the most commonly employed approach, with most patients experiencing significant neurological improvement after complete removal of the migrated fragment (6, 12). To summarize previously reported treatment strategies and outcomes, representative studies from the literature are listed in Table 1.

**Table 1:** Reported cases of tumor-mimicking migrated disc fragments and treatment outcomes.

Author	Year	Location	Treatment strategy	Outcome
Hoch & Grinberg (8)	2010	Lumbar spine	Open surgical excision	Complete symptom resolution
Akhaddar et al. (3)	2011	Lumbar spine	Laminectomy/hemilaminectomy	Neurological improvement
Stavrinou et al. (9)	2011	Cervical spine	Conservative treatment	Rapid spontaneous recovery
Jia et al. (10)	2018	Lumbar spine	Surgical excision	Favorable recovery
El Asri et al. (11)	2008	Lumbar spine	Open posterior decompression	Marked symptom relief
Elsharkawy et al. (6)	2019	Lumbar spine	Predominantly surgical (review)	Good neurological outcomes in most cases
Hamid et al. (12)	2025	Thoracic/Lumbar spine	Predominantly surgical	Sustained symptom improvement

The Quadrant tubular retractor system was developed as an advancement in minimally invasive spine surgery, aiming to reduce muscle dissection, preserve posterior spinal structures, and minimize postoperative pain while maintaining adequate visualization of neural elements. Compared with traditional open laminectomy, tubular approaches enable targeted exposure via a muscle-splitting corridor, thereby decreasing blood loss, lowering the risk of postoperative instability, and shortening recovery time. For the management of posteriorly migrated disc fragments, the Quadrant approach offers carries several theoretical and clinical advantages. First, this approach provides sufficient operative space to safely identify and resect posterior epidural lesions while limiting unnecessary bony resection. Second, the enhanced illumination and magnification facilitate meticulous dissection around the dura mater and nerve roots, which is particularly critical when preoperative imaging indicates a tumor-like lesion. Third, preservation of posterior spinal structures may reduce the risk of postoperative spinal instability, especially in elderly patients with pre-existing degenerative changes (6, 12). Despite these compelling benefits, reports on the application of the Quadrant system for treating tumor-mimicking posteriorly migrated disc fragments remain scarce in the literature. As a single-case report with 6-month follow-up, larger series are needed to confirm long-term stability and recurrence rates.

In the present case, heightened awareness of this rare clinical entity is crucial for optimizing differential diagnosis and guiding rational treatment strategies. For patients with progressive neurological deficits and inconclusive imaging findings, surgical exploration remains a reliable strategy to establish a definitive diagnosis and effectively decompress neural compression. The Quadrant minimally invasive approach permits complete excision of the lesion while minimizing surgical trauma, thereby facilitating rapid symptomatic relief and favorable short-term clinical outcomes. Additionally, this technique helps preserve spinal stability by maximizing the retention of normal anatomical structures and reducing the potential risk of injury to neural elements and the dura mater.

## Conclusion

PEMDF represents a rare but important tumor-mimicking entity that poses significant diagnostic challenges on MRI, particularly in the presence of rim enhancement and posterior extradural localization. This case demonstrates that a Quadrant-based minimally invasive approach enables precise posterior epidural access, safe microsurgical dissection around the dura and nerve roots, and complete fragment excision with minimal bony resection. Compared with conventional open laminectomy, this technique preserves posterior spinal elements, reduces surgical morbidity, and facilitates rapid neurological recovery while maintaining diagnostic accuracy through histopathological confirmation. Clinically, our findings support minimally invasive tubular approaches as a valuable alternative surgical option for selected patients with progressive neurological deficits and indeterminate imaging, thereby addressing diagnostic uncertainty with effective and tissue-sparing management. Further investigations are warranted to validate long-term outcomes and establish optimal patient selection criteria.

## Data Availability

The original contributions presented in the study are included in the article/Supplementary Material, further inquiries can be directed to the corresponding author.
